# Supplementary material for: Patterns of Intron Gain and Loss in Fungi
Source: PLoS Biol. 2004 Nov 30;2(12):e422. doi: 10.1371/journal.pbio.0020422 (PMC532390; doi:10.1371/journal.pbio.0020422)
Supplement: Table S1 — Also available at http://genes.mit.edu/NielsenEtAl/. (4.3 MB ZIP). [file pbio.0020422.st001.zip › NielsenEtAl/html/233.html]

AN3620.1.NCU00942.1.MG03676.1.FG00281.1


```
 CLUSTAL W (1.82) Multiple Sequence Alignments - Introns Inserted


Sequence 1: MG03676.1	338 aa
Sequence 2: FG00281.1	437 aa
Sequence 3: NCU00942.1	451 aa
Sequence 4: AN3620.1	396 aa
Alignment Length: 549 aa
Number Identitical Residues: 89 aa
Alignment Score (without introns) 5759


MG03676.1 	----~-------------------------------------------------------
NCU00942.1	----~--------------MDSPLIEVWHAASGSPFLPTVGKGTQFFVASLLLLIGFSLT
FG00281.1 	MRHH1QHQLPKLTSFPTKPMDSSLHEVWQAAAGSPFFPTVDKDTQFTLAFALLLVGFFLT
AN3620.1  	----~-------------------------------------------------------
          	                                                            

MG03676.1 	------~------------------~-------------------------------MAD
NCU00942.1	GAFTLN1RSIVNLLLLGIPASLAFA2SS----------------------ASLASN--MS
FG00281.1 	GVFALN1RTLINVPLLGIPASLTLA2WSNQEKTLLGAPIAFLPVFEPFIHAPFPNNHIRN
AN3620.1  	------~------------------~----------------------------------
          	                                                            

MG03676.1 	SARSQPGPGVFRAVTSSTRPLHQLLRAISFTNKIHVEISEDGIRFTAEHARVMQG1VASL
NCU00942.1	QPTQDAGPPLFRAVASSTRPLYQLLKAVGFTNKIHVEITPEGLRFSADHARVMQG~AAHW
FG00281.1 	FDMSTEAQPIFRAVATSTRPLYQLLRCINFAPRVHVQITEEGIRFASDHARVMQG1VAFL
AN3620.1  	---MTDTGPIFTAVSSNANQLYTLLHCISFAQNATVQITPDGIRFSVEEGRVVQG~LAFL
          	         :* **::.:. *: **:.:.*: .  *:*: :*:**: :..**:**  *  

MG03676.1 	NKALFSSFVLNLPE-AAAQGGEEYENTPV---VPNFQISLPALIETLQLFGLADAAVRQA
NCU00942.1	NKNLFASYTTNLPP-AAGNGDDD-DPEPE---PVSFQISLPAFLETLQIFGVADVAARQA
FG00281.1 	DKSLFSSYTVNLPS-QDG------DDAPE---LPNFQLSLSSVLEVLQIFGAVDVATRAH
AN3620.1  	DKALFTTYTFHPPTGVNNEHDTFMDTEPDGANYPCFVVSLSALLETLKIFGIGDSSSAST
          	:* **:::. : * .   . .   :  * .:.   * :**.:.:*.*::**  * :    

MG03676.1 	KTEADP-YRSNLRN---YR----PDAFSNQTLG--------VAGTCCLSYSEEGAPFSIN
NCU00942.1	RSDIDPLVRGNPRMNHQHRGGNGSDAFSHQTLHGGGFASSSSNGTCILSYPSDGGPFSII
FG00281.1 	KAEQDP-YRSNLRN---YR----PDAFSNQTLG--------ISGTCTLLYAEEGDPFKIT
AN3620.1  	SRAASVQPPTASAS----------NAFTAPALL--------LNRSCTFQYLTDGSPLTVT
          	    .                   :**:  :*            :* : *  :* *:.: 

MG03676.1 	MEEAGVNTTCNLVTYLP-DAPEDIPFDREGISFKIIMQSRWLLDALAELAPTSPNRLTIT
NCU00942.1	LEEAGVKTTCNLTTYLP-EMPEDIPFDIDDLSFKVIMQARYLLDALAEVGSINPERLTIV
FG00281.1 	IEESGVKTTASLTTYLP-EIPDDIPFDRTDLTFKIIMQSRTLLDSLAEISPTAPLKLTIT
AN3620.1  	LTETGVKTVCELTTYEPDEGDLEIPFQRDGIVMKIIMRSAWLHNAITELGATNPSILKIS
          	: *:**:*...*.** *.:   :***:  .: :*:**::  * ::::*:..  *  *.* 

MG03676.1 	AS-KREPFLRLSGSGPLG-------SSSVDFSRGRD------------------------
NCU00942.1	VS-RKEPYLRFRTSGSHSGAGGPGVESTVDFRRGRD------------------------
FG00281.1 	AT-KASPFLSLAGAGDLG-------SSGVDFARGRD------------------------
AN3620.1  	ASDKQEPYFTLSGAGGPFS------ESTVEFSIEQDEGRKGTTHDTHRKVMTNDGARPRA
          	.:.: .*:: :  :*   .      .* *:*   :*.. ..:: .:  .  :...: . :

MG03676.1 	--------LLETFSVR--------ERWAQSFKFDIIKSASEAMRISSKVSLRGDGQGVLN
NCU00942.1	--------LLETFSVRAVERAKGERRWSQTFKFDTIKAATEAMRIASKVSLRGDGQGVLS
FG00281.1 	--------LLETFTIQ--------DRWSQAYKFDFIKNSTEAMRIANKVSLRGDSQGVLS
AN3620.1  	TRTKLAPTVTETFLISPPSSMG--SRLRQDFRFAYIQKAARAMATANKVSIRGDRQGVLS
          	: :. :.:: *** : . .      *  * ::*  *: ::.**  :.***:*** ****.

MG03676.1 	FQFMVEVEGGG----------------------------MQWLEFRFVPYLTREDDEEDD
NCU00942.1	MQFMVEVEGAGGAGGG----------------VPGGGAGLSFLDFRFVPYATDGDEETDT
FG00281.1 	LQFMVDIEGGK----------------------------RSFLDFRFVPFLQEDGEDDDT
AN3620.1  	LQFMIEFDASGSAMTGNSVGNSMRSGAVRGVDVNAPGSTVSFVDFRFVPLLDEDDLGGKA
          	:***::.:.. .:  ..: ..:  :.:  . .  . .:  .:::*****     .   . 

MG03676.1 	AAAEDDEEEDDQE
NCU00942.1	EGEGDDVDMSEA-
FG00281.1 	DGAE---------
AN3620.1  	VRDDLE-------
          	     .
```
